# Retrospective analysis of common diseases in pediatric oral emergency patients of Ningbo

**DOI:** 10.3389/fdmed.2025.1533546

**Published:** 2025-08-25

**Authors:** Jun-ji Xu, Lan-qiu Lv, Shanshan Guo

**Affiliations:** ^1^ Department of Oral Surgery, Affiliated Women’s and Children’s Hospital of Ningbo University, Ningbo, Zhejiang, China; ^2^ Department of Child Health, Women’s and Children’s Hospital, Ningbo University, Ningbo, Zhejiang, China; ^3^ Department of Otorhinolaryngology Head and Neck Surgery, Ningbo Yinzhou No. 2 Hospital, Ningbo, Zhejiang, China

**Keywords:** children's oral emergency, disease type, patient volume, pediatric dentistry, pedodontics, visit period

## Abstract

**Background:**

With the increase in the resident population and the number of children in Ningbo, as well as the growing demand for oral health care, the number of children's dental emergencies has been increasing year by year. This trend not only increases the pressure on medical resources, but also puts higher demands on the level of children's oral emergency care. In order to better guide the diagnosis and treatment of common and frequent diseases in paediatric oral emergency care, and to improve the efficiency of the use of medical resources. A retrospective report is needed to provide a valuable reference for improving the level of paediatric oral emergency care.

**Objective:**

To study the morbidity characteristics of children's oral emergencies. Through the clinical data of children's oral emergency cases in our hospital during the period of 2015–2021, we analysed the types of oral emergency cases, gender, age, and time of injury visits, so as to better guide the diagnosis and treatment of common and frequent diseases in children's oral emergency.

**Methods:**

A total of 4,946 cases of dental emergencies attended between January 1, 2015 and December 31, 2021 were collected and statistically analyzed in a retrospective cohort with respect to gender, age, time of visit and diagnosis of the patients. SPSS 19.0 software was applied to test and analyze the results.

**Results:**

There were 3,096 males and 1,850 females in 4,946 children's oral emergency patients, with a male to female ratio of 1.67:1; The average age was 3.9 years old. The highest number of children's oral emergency visits was October, the lowest was January, and the highest time was 20:00–21:00; The first three diseases in emergency diagnosis were maxillofacial trauma (60.17%),dental trauma (17.19%) and acute periapical periodontitis (13.26%).

**Conclusion:**

The number of pediatric dental emergencies has been increasing year by year, from 298 cases in 2015 to 1,827 cases in 2021, with a relatively high concentration of visits and a wide range of emergencies, including: maxillofacial trauma, dental trauma, acute periapical inflammation, acute endodontitis, and oral aphthous ulcers, but with a major focus on maxillofacial trauma (60.17%) and dental trauma (17.19%). Therefore, emergency management of maxillofacial trauma by pediatric emergency dentists is a clinical priority in pediatric emergency dentistry.

## Introduction

In dental medicine, children's oral emergencies are of utmost importance and a core focus in dental clinics. These emergencies cover a broad range of issues, including injuries and inflammation of oral and maxillofacial bones, joints, teeth, and soft tissues (1, 2). They are marked by a large patient volume, acute onset, and complex conditions. Children's limited self—protection skills, due to their young age, result in a high frequency of oral emergencies. Their active and inquisitive nature makes them more prone to accidents. Additionally, the diagnosis, treatment, and prognosis of children's oral problems differ from those of adults. Diagnosis can be difficult as children may struggle to describe their symptoms accurately. Treatment requires careful consideration of a child's cooperation ability, the impact on dental and jaw development, and proper use of anaesthesia. Prognosis must account for long—term effects on oral health and overall growth.

To understand the characteristics and patterns of paediatric oral emergencies in our hospital, a retrospective analysis was carried out. The study focused on cases from 1 January 2015–31 December 2021. This period was chosen to obtain a substantial number of cases for trend and pattern identification. The analysis aims to enhance our understanding of the epidemiology, causes, and treatment outcomes, enabling us to develop better strategies for diagnosis, treatment, and prevention, thus improving children's oral health.

### Information and methodology

#### Clinical information

A total of 4,946 cases who visited our dental emergency department from January 1, 2015 to December 31, 2021 were collected.^1^ They were retrospectively analyzed for type of disease, gender, age, and time of injury visit.

#### Research methods

A retrospective cohort study was used to analyze the data, and all dentists participating in the program received uniform training to ensure consistency and accuracy of data collection. The data for the study was obtained from the doctors' workstations, where the patients' gender, age, time of visit and diagnostic information were recorded. Patients were categorized into five age groups: ≤1 year old, 1–3 years old, 3–6 years old, 6–12 years old, and 12–15 years old, in order to observe the morbidity characteristics of children's oral emergencies in different age groups; and the time of consultation was grouped according to year, month, and time period. The year was used to analyze the trend of the number of children's oral emergencies each year; the month was used to explore the differences in the number of emergency cases between different months; the time period was further subdivided into each hour, such as 00:00–24:00, to clarify the peak and trough times of emergency visits in a day; and the diagnosis was made in accordance with the international classification of diseases (ICD-10), so as to observe the incidence characteristics of children's oral emergencies in different age groups. diseases (ICD-10) (3).

Excel 2013 was used to filter and sort the data, and the count data were expressed as the number of cases (constitutive ratio), so as to visualize the distribution of the data in each category (4). SPSS 19.0 software was used to test and further statistically analyze the data to test whether the difference between different age groups was statistically significant (*P* < 0.05) to determine whether the difference in the distribution of different age groups in pediatric oral emergencies was significant.

### Dispatch

#### Age and gender distribution

Among the patients in this group, 3,096 were male and 1,850 were female, with a male to female ratio of 1.67:1. The top 3 age groups for pediatric oral emergencies were 1–3 years (34.88%), 3–6 years (27.46%), and ≤1year(19.75%).The difference between the groups was statistically significant (*P* < 0.05), as shown in Table 1.

**Table 1:** Gender and age distribution of patients.

Group	Gender		Consider
	Male	Female	
≤1 year old	593	384	977
1–3 year old	1,073	652	1,725
3–6 year old	863	495	1,358
6–12 year old	528	295	823
12–15 year old	39	24	63
Consider	3,096	1,850	4,946

#### Time of illness onset

**Figure 1:**
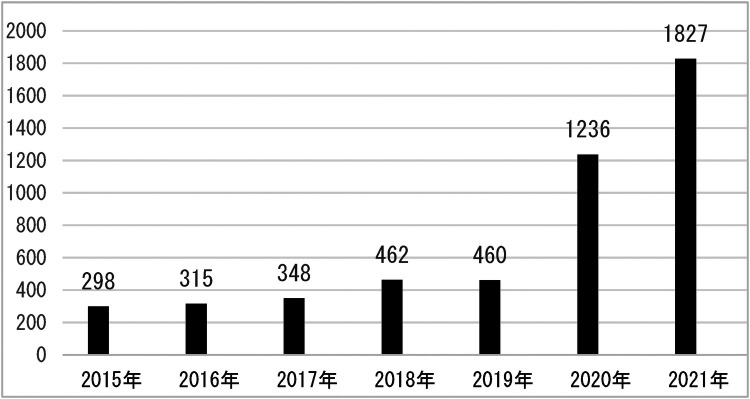
Distribution of oral emergency patients in each month from January 1, 2015–December 31, 2021.

The volume of pediatric oral emergencies for each year from 2015–2021 is shown in Figure 1.

As shown in the figure, with 298 cases in 2015 and up to 1,827 annual oral emergencies through 2021, there is a clear trend of increasing pediatric oral emergencies from year to year, with the greatest increase especially between 2020 and 2021.

**Figure 2:**
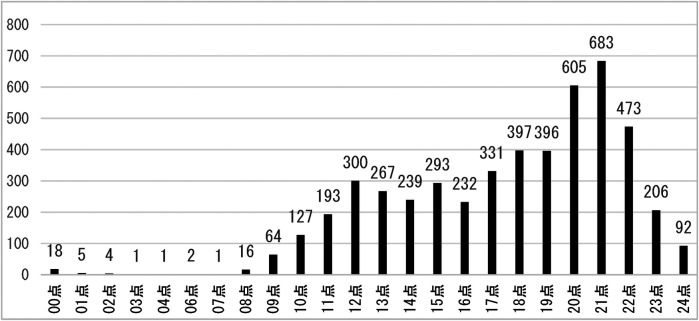
Distribution of oral emergency patients in each time period.

The volume of emergency room visits by time period is shown in Figure 2.

The above graph shows that the volume of pediatric dental emergency patients is relatively low from 0:00–6:00, and then begins to rise gradually until 11:00–12:00, reaching a small peak; then falls back to 15:00–16:00, reaching a small trough, and then gradually rises until 20:00–21:00 to reach the highest, and then falls back.

**Figure 3:**
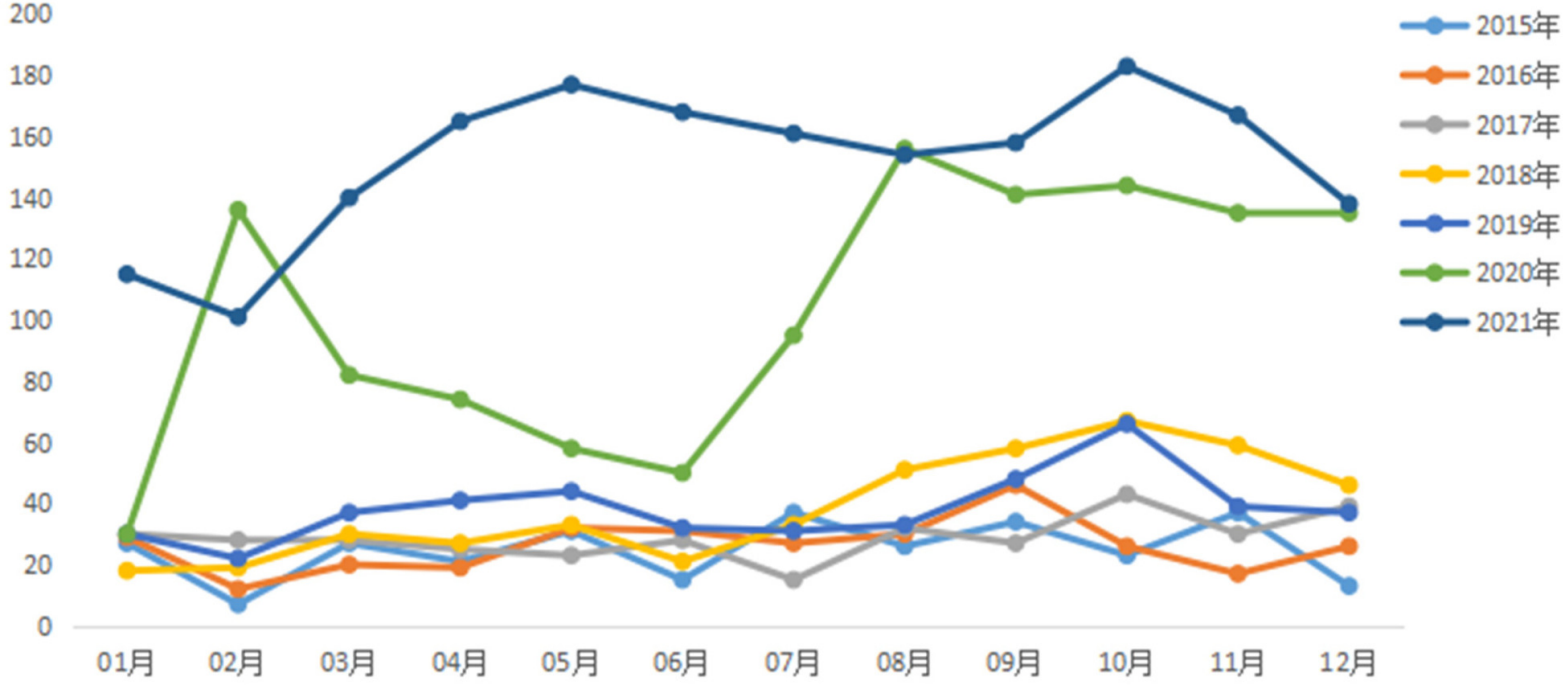
Distribution of oral emergency patients in each month from January 1, 2015–December 31, 2021.

Oral emergency room visits by month from 2015–2021 are shown in Figure 3.

As shown in the graph October was 552 cases higher than other months and January was 279 cases lower than other months.

#### Analysis by diagnosis

According to the situation of children's oral emergency patient consultations in the past few years, it can be summarised into 9 categories of diseases. Of these, 60.17 per cent of children's dental emergencies were dominated by maxillofacial trauma, followed by dental trauma and acute periapical inflammation, accounting for 17.19 per cent and 13.26 per cent, respectively. Other details are given in Table 2.

**Table 2:** Analysis of types of oral emergency diseases.

Diseases	*n*	%
Maxillofacial trauma	2,976	60.17
Dental trauma	850	17.19
Acute periapical infection	656	13.26
Acute pulpitis	180	3.64
Oral aphthous ulcers	154	3.11
Maxillofacial infections	93	1.88
Acute parotitis	14	0.28
Fracture of the mandible	10	0.2
Acute pericoronitis	7	0.14
Other	6	0.12

### Discussions

Oral emergency room refers to patients who come to the clinic urgently due to sudden onset of acute oral and maxillofacial pain, bleeding, swelling and various causes of dental and periodontal tissue injury and other unforeseen events. Oral contingencies are more common among children due to various reasons such as their young age, immature mental development and lack of self-protection awareness in life activities (5, 6). Oral and maxillofacial region is rich in blood, trauma bleeding is more, once the child has an accident, bleeding is more, most of the children suffer from more than one, the parents of the children are therefore worried and anxious, and the willingness to seek medical treatment is very urgent.

The results of the present study showed that oral emergencies in children were predominantly in the age group of 1–3 years (34.88%), with a male to female ratio of 1.65:1, which may be related to the fact that boys are more mobile, adventurous and aggressive at this stage. After the 1–3 years age group, the 3–6 age group follows (27.46 per cent), with a male-to-female ratio of 1.74:1. At this stage, young children are inquisitive, have a large volume and range of activities, but their mobility and coordination of their limbs are poor, and their sense of protection and ability are not sound, making them the most vulnerable to injury (7). The number of dental emergency room visits has risen each year from 2015–2021, with a significant increase of 776 visits by 2020 and the most in 2021. This may be related to the increase in the resident population and the number of children in the Ningbo area in recent years in the local area, and it may also be related to the advancement of our diagnostic and treatment technology and the increase in the people's trust in our hospital as well.

The study showed that October 2021 was the peak month for oral emergencies, which accounted for an average of 11.16% of annual emergencies, which is in general agreement with the study by Yan Liu and Xiao Xu et al. (8, 9). This may be related to the fact that the parents of the children were busy with their work and chose to concentrate on outings during the “11th November” holiday period, which increased the risk of traumatic injuries. The study found that February 2015 was a low point for visits, possibly due to the cold season, when people have fewer opportunities to go outside and are at a lower risk of trauma, and also due to the decrease in the urban residential population during the Chinese New Year period, which would result in a corresponding decrease in the number of emergency dental care visits. Analysing the statistics of the results of this study by time of the day, night time (20:00–21:00) is the peak time period for emergency care, which is related to the activity of the residents after dinner and before bedtime activity time period, thus increasing the incidence of dental and maxillofacial trauma. According to the characteristics of the emergency peak period, hospital management staff should pay attention to the graded attendance of dental emergency patients, so that emergency patients get timely diagnosis and treatment, and reduce the waiting time of patients. At the same time, in the midday, night, national holidays and other patients with a high volume of emergency treatment period, should implement the number of health care personnel to work and shift preparation system at the same time to formulate a comprehensive children's dental emergency emergency contingency plan.

This study found that the incidence of oral emergencies in children showed an increasing trend between 2015 and 2021, which is consistent with the findings of a study by Yiming Yu et al. (10) from the School of Public Health, Fujian Medical University, published in the Chinese Journal of Stomatology 2023. The study noted that the disease burden of adult periodontal disease in China is increasing and the incidence of periodontal disease may continue to grow in the next 25 years. This suggests that the incidence of oral emergencies in children is also likely to increase further as the population grows and oral health problems become more prominent.

In terms of the types of children's dental emergencies, the top three were maxillofacial trauma (60.17 per cent), dental trauma (17.19 per cent) and acute periapical inflammation (13.26 per cent), in that order. The maxillofacial region is rich in blood vessels and bleeds more, coupled with the lack of children's own ability to express themselves, and more do not cooperate with the diagnosis and treatment, so children's oral emergency physicians should be more comprehensive and careful in order to prevent missed diagnosis and misdiagnosis. JIN Huang, LI Junyan et al. concluded that the therapeutic management of severe pediatric maxillofacial trauma should begin with assessment of vital pointers, emergency hemostasis, and further specialty management when the general condition is stabilized (11, 12).

Dental trauma typically manifests itself in the form of tooth concussion, tooth loosening, and tooth dislocation. This is in general agreement with the study by Zeng Jiahao et al. (13). Emergency paediatric dentists should focus on the management of traumatic dental injuries so that children can receive timely and standardised treatment to maximise the retention of affected teeth. According to Theoloqie-Lyqidakis et al. (14, 15), accurate diagnosis, prompt treatment and follow-up are essential for the prognosis of alveolar trauma in children.

This study shows that acute periapical infections are the third most common in the region, which may be related to the difficulty in accessing dental care and the prolongation of treatment appointments in the context of the growing demand for dental care. Of course, there is also a correlation with the fact that some parents still believe that milk teeth need to be replaced and do not need to be treated, thus preventing caries from being treated in a timely manner and allowing it to develop into acute periapical infections. This suggests that future research should pay more attention to children's oral health education and family oral health behavior intervention. Therefore, the prevention and treatment of dental caries is still a major concern in our clinic.

## Conclusion

To summarize, children's oral emergency consultation time is relatively concentrated, but the complexity of trauma and the wide range of diseases, children's oral emergency physicians should have solid theoretical knowledge, comprehensive diagnostic and treatment techniques and a high level of clinical emergency response ability, so that patients can be efficiently rescued and treated. At the same time, the future should also strengthen the daily oral mission to improve the public's awareness of accident prevention and help improve emergency treatment capabilities is also very necessary.

## Footnotes

^1^All study participants, or their legal guardian, provided informed written consent prior to study enrollment.
